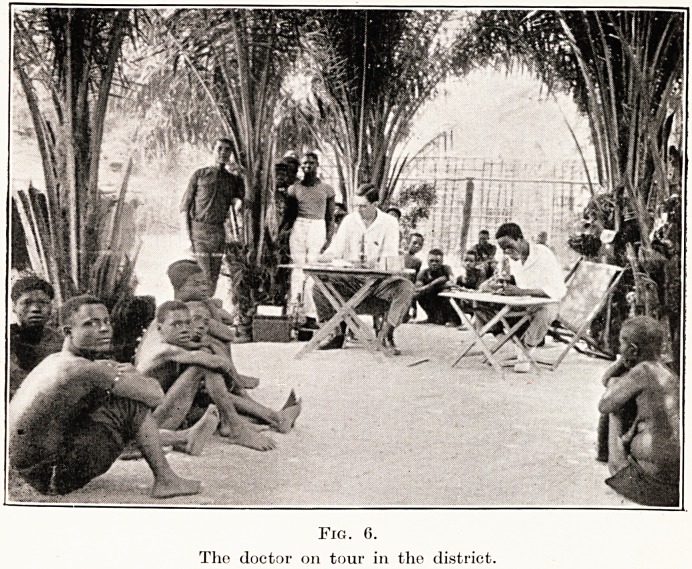# The Twenty-Eighth Long Fox Memorial Lecture: Forest Folk: Modern Medicine in the Congo

**Published:** 1939

**Authors:** C. C. Chesterman


### The Bristol Medico-Chirurgical Journal

*" Scire est nescire, nisi id me*
*Scire alius sciret."*

WINTER, 1939.

### THE TWENTY-EIGHTH LONG FOX MEMORIAL LECTURE:

BY

DR. C. C. CHESTERMAN, O.B.E., M.D., M.R.C.P.*

DELIVERED IN THE UNIVERSITY OF BRISTOL
ON TUESDAY, 14th NOVEMBER, 1939.

THE VICE-CHANCELLOR (Dr. T. LOVEDAY, M.A., LL.D.)
*in the Chair.*

ON

### FOREST FOLK: MODERN MEDICINE IN THE CONGO.

THE subject upon which I propose to speak seems particularly appropriate, for Dr. Long Fox was always especially interested in the foreign mission field. Indeed, it is to remind us of this lifelong interest that the Long Fox Memorial Fund has the dual purpose of providing for this lecture and of helping students who are preparing for medical missionary work. As an old student of this University and one of the many from

* Owing to the regrettable absence of Dr. Chesterman the lecture was read by Dr. Stanley G. Browne, M.R.C.P., F.R.C.S., of Yakusu, Congo Belge.

R

Bristol who have carried modern medicine to forest folk and other primitive peoples, I esteem it a very great privilege to be asked to deliver this lecture, and hope that in commemorating a great man it will stimulate others to cherish his ideals. I intend to talk about forest folk and modern medicine, not in two sections, but in the contact between them which it was my privilege to be one of the first, in a large area, to effect.

The Belgian Congo has an area of one million square miles, situated across the Equator in the west-central region of Africa. In density of population, with 10 to a square mile, it comes somewhere between Tanganyika with 4·5 and Nigeria with 22 to the square mile. There is therefore no population pressure problem, which so appals the medical and hygienic worker in some parts of the East. The country could well support ten times the number of inhabitants.

The central area is one dense forest intersected by ten thousand miles of navigable waterways and hundreds of thousands of miles of streams. For many years it shared with the West Coast of Africa the evil reputation of being the "white man's grave": and during the first ten years of the history of many missionary societies operating in the Congo basin 50 per cent. of the personnel died or had to be invalided home without completing one term of service. It must be remembered that in 1878, the year after Stanley completed his 999 days' journey from east to west and emerged at Boma, hardly any pathogenic germ had been recognized under the microscope. The ætiology of malaria was quite unknown. Fevers were described by their symptoms, quartan, tertian, remittent, bilious, etc., and death from malaria was generally attributed to sun fever, brain fever, or meningitis; generally the end was hastened by giving calomel in larger doses than quinine. The rôle of arthropod vectors in human disease had not been suspected, nor had the various forms of dysentery been differentiated. It had not yet been realized that the hookworm was responsible for African lethargy, and the pathology of sleeping sickness was still a profound mystery.

There are now very few tropical diseases which have not yielded their secrets to the researches of science, and chemists are yearly increasing our armamentarium of synthetic drugs with which efficiently to treat them. But the acquisition of knowledge has proceeded much quicker than the application of it, and it is this time-lag which a physician in the forests finds it his duty to endeavour to reduce. It is still a fact that 100,000 people die of sleeping sickness in tropical Africa every year, and malaria is still probably the most killing disease among children, where infant mortality frequently reaches figures greater than 500 per 1,000.

The region which we consider is that of the neighbourhood of Stanleyville, where the Congo river takes its great westward bend. (Fig. 1.) In the tropical forest the temperature in the shade varies from 65 to 85 degrees, but the relative humidity is so very high that at least 20 per cent. of one's energy is consumed in the active process of sweating. The peoples belong to the Bantu family, but are broken up into innumerable small tribes which have remained isolated through mutual suspicion. They have never had to co-operate for agricultural purposes or for defence, which are the commonest stimuli to civilization among other races.

There is very little demarcation of the seasons, which are recognized more by the level of the river than by a changing climate: and therefore there has been little leisure for the development of the arts. The riverine folk are occupied mainly in fishing and trading, living for many months of the year in their large canoes, while the forest tribes spend their lives in hunting and the growing of vegetable produce—cassava, plantains, etc. (Fig. 2.) The pygmies live in groups with no fixed abode and rarely come into contact with the more settled inhabitants. They are expert huntsmen and exchange their meat for vegetable produce at recognized rendezvous, often without actually coming into contact with the villagers.

Inter-tribal life is organized on a basis which is partly self-sufficient and partly co-operative, fish being exchanged for meat and vegetable produce for palm oil, salt, etc. It is a most interesting experience to accompany a hunting party in the forest, and to take one's place, hidden behind a large tree, a few yards in front of a row of nets fixed breast high and extending for a kilometre or more. The Congo hunting dogs, which are about the only dogs in the world which do not bark, drive the game towards the nets by the noise of the wooden rattles fixed round their necks. When an antelope or wild pig careers madly past an effort is made to spear it or to transfix it with an arrow before it gets entangled in the meshes of the net, where one is expected to hold it by horns or tail till help arrives.

Elephants abound and are very destructive, but perhaps the most interesting denizen of the forest is the chimpanzee. These beasts attain a great size, weighing over eighteen stone, and will sometimes attack women coming home from the garden if they are carrying baskets of food on their backs. We have had a number of patients in hospital who have had bones broken in an unequal encounter with a chimpanzee. One man who tried to revenge himself for the injury to his comrade, whose gun the brute had snatched from him, climbed a tree, armed only with a knife: but the chimpanzee descended, took him by the arm, swung him round and dropped him to the ground with a fractured humerus. Natives believe that they are town folk who have done evil, thus favouring the theory of devolution rather than evolution! My hospital hunter used frequently to bring them in as food for patients and recounted stories of how they would plug their wounds with leaves and shout at him when wounded.

One often gets the impression that the primitive forests belong to the animal and insect world and that man only lives there on sufferance. The native, however, thinks of his great "Mother Forest" as the inexhaustible store of material and of food and the safe retreat in times of danger.

A medical man soon comes up against a wide variety of surgical and medical diseases. The former are perhaps more spectacular. Among people who are entirely naked or for whom "full dress" is about the size of a post card, many deformities from injury or scars from burns are to be observed. Tattooing of the body is universal among women, but as many of them have a tendency to keloid formation, the scars become very unsightly by our standards. An umbilical hernia is looked upon as a mark of beauty and is sometimes deliberately produced in small children. The piercing of the lobe of the ear and insertion of larger and larger objects enables a teacup to be lodged in the resultant fibrous loop without difficulty. Similarly, pieces of ivory, often as big as a five shilling piece, are worn in the upper lip. This custom has a curious influence on language in some parts, where "W's" are substituted for "B's," the pronouncing of which becomes rather awkward with an upper lip hanging like a curtain over the mouth.

Tumours of every type and site are met with, the commonest innocent one being lipoma. Melanotic carcinoma of the palm or sole is in my experience the commonest malignant tumour. This is curious since neither the palm nor the sole of negroes is pigmented. Possibly the chronic fissures of crab yaws may be a contributory factor in their formation. Carcinoma is not infrequently met with, but the fact that the average duration of life is much less than in civilized countries may account for the relatively lesser incidence.

Ulcers of various kinds are very common, and chronic ulcers frequently become epitheliomatous. The tropical ulcer caused by the same organisms as are found in Vincent's angina is very liable to attack weakly or ill-nourished victims whose superficial tissues are rapidly destroyed. The incidence of peptic ulcers is very low in the region under consideration, as is also that of appendicitis. In fact, I was never really certain that I was dealing with a case of either. In India duodenal ulcers are 600 times more common in the South than in the wheat-eating North : the ætiology seems to be connected with a diet deficient in vitamin A. On this hypothesis one would expect these diseases to be rare in tropical Africa, where large quantities of red pepper and red palm oil, both rich in vitamin A, are consumed.

The most spectacular surgical condition is that of elephantiasis, and many victims drag themselves round with what a colleague facetiously called "spare parts." So massive may elephantoid tumours become that it is not infrequently necessary to remove the man from the tumour rather than *vice versa.*

Medical diseases are, however, of more importance from a public health point of view. The primitive forest is the parasites' paradise. Practically everybody is a walking pathological museum and harbours a dozen or more of the following varieties of parasites :—

| | | |
|---|---|---|
| BLOOD AND LYMPH. | | |
| | Malaria (four varieties) : | *P. falciparum.* |
| | | *P. vivax.* |
| | | *P. malariae.* |
| | | *P. ovale.* |
| | Sleeping Sickness : | *T. gambiense.* |
| | | *T. rhodesiense.* |
| | Filaria (four varieties) : | *L. loa.* |
| | | *A. perstans.* |
| | | *O. volvulus.* |
| | | *W. bancrofti.* |
| SKIN. | | |
| | Leprosy : | *M. leprae.* |
| | Yaws : | *T. pertenue.* |
| | Ringworm (two varieties) : | *T. flava.* |
| | | *T. cruris.* |
| | Scabies : | *S. scabiei.* |
| | Jiggers : | *T. penetrans.* |
| | Myiasis (various). | |
| INTESTINES. | | |
| | Nematodes : | *A. lumbricoides.* |
| | | *A. duodenale.* |
| | | *N. americanus.* |
| | | *S. stercoralis.* |
| | | *T. colubriformis.* |
| | | *T. deminutus.* |
| | | *T. dispar.* |
| | | *E. vermicularis.* |
| | Trematodes : | *S. mansoni.* |
| | | *S. haematobium.* |
| | | *S. intercalatum.* |
| | Cestodes : | *T. saginata.* |
| | | *H. deminuta.* |
| | | *Sparganum mansoni.* |

The above is not a complete list of the commoner parasites more or less special to the region, but in addition to these there are the cosmopolitan scourges including venereal diseases and tuberculosis, both of which are increasing in alarming proportions.

It will be seen that no man liveth unto himself in the Congo, and if there is any justification in the title "lazy nigger" it is to be found in the fact that he has to give board and lodging to a vast number of uninvited guests.

Yaws is at once a calamity and benefit to forest folk: a calamity because it causes so much misery among the children in the secondary stage and so much ulceration and deformity in the tertiary stage, but a blessing in that its easy and effective cure has established the reputation of modern medicine. In fifteen years above 30,000 cases were treated at Yakusu and its surrounding dispensaries by combinations of "914," bismuth and stovarsol.

Leprosy is found to be affecting between 0·5 and 2 per cent. of the population; but in the absence of clothes and with a fairly liberal diet and outdoor life it does not as a rule progress rapidly to the more destructive type of disease seen in other lands. It is interesting to note that natives had always distinguished the more chronic tuberculoid variety from the lepromatous type, a distinction only recently made in medical terminology. The disease is not very much feared and segregation is not insisted upon, which accounts for the continued infection of children. This, as in the case of so many diseases, keeps the disease going.

Sleeping sickness, about which we shall say more later on, was at one time a menace to the district, and miserable was the lot of those sufferers who became incapable of helping themselves and were turned out into the forest, to die or to be taken by wild animals.

This brief recital of the local pathology is sufficient to convince you, as it did us, that if any good is to be

### PLATE V

Fig. 1.
The Belgian Congo.

Fig. 2.
A hunting family.

### PLATE VI

FIG. 3.
Making bricks.

FIG. 4.
The hospital at Yakusu.

done a serious attempt is needed and a well-organized work must be established. Our first objective was, therefore, the erection of a base hospital. A suitable piece of ground was obtained on the site of a former defensive stockade, and the work of making and baking bricks was commenced. (Fig. 3.) The clay thrown up by white ants is excellent material for bricks, and the blue clay from the river bank, used by native women for making their cooking pots, is suitable for the manufacture of tiles, which are burnt in rows between the bricks in the kiln. Mahogany and other hardwood trees were felled in the forest and cut up by gangs with long pit-saws. Masons had to be trained to bond and lay bricks, for no contractors were available.

A comfortable and permanent doctor's house was the first necessity and cost about £600 to erect with corrugated iron roof. While foundations for the administrative block of the hospital were being laid it was noticed that women going to market would not pass by, but made a long détour to avoid approaching the building. It was discovered that this was on account of the existence of inner rooms, which they imagined were for the purpose of canning meat which was to be hacked off any patients who were foolish enough to enter the hospital portals !

The completed block (Fig. 4) comprises waiting-room, lecture-room, laboratory, dispensary, X-ray room, nurses' room, theatre, and sterilizing room. The whole unit of about seventy-five beds includes wards for men and women, private wards, and a *Maternité Reine Astrid* in memory of the late Queen of the Belgians, so tragically killed. A white patients' block, kitchen and laundry, and stores were also provided. One of the wards was given by the Baptist Churches in Bristol, which name it bears. Beds are of the Lawson Tait pattern with one-inch felt mattresses enclosed in calico cases. These mattresses can be washed with disinfectant and easily dried in the sun. Pillows are filled with local kapok. Electric light has been installed throughout, and rain-water is collected from the roofs and pumped to an elevated tank.

The operating theatre is in constant use, the majority of operations being done under spinal anæsthesia (stovaine) which has proved entirely successful and satisfactory in some thousands of cases. The native calls the surgeon " one who cuts up some and divides others." The windows are usually left open for spectators to appreciate that there is more method than magic in surgery. Native surgery is restricted almost entirely to the operation of circumcision, and only one instrument, the knife, is required for this. They are very modern, however, in preferring an operating-theatre to be at the top of the building, free from dust and with ample air and light. The operation of circumcision is frequently performed on a platform raised fifty feet from the ground. It is an ordeal and trial of courage for the young initiates, who have to go through the rite in full gaze of the public.

One comes up more against the native witch-doctor than the native surgeon: for among primitive animists the whole of pathology is reduced to the simple formula " witchcraft." The question is not asked: " What caused the disease ? " but " Who ? " Folk argue by the simple method of analogy—if I have an enemy I want to do him harm: therefore when harm comes to me my enemy has done it. The witch-doctor is not Public Enemy No. 1, but the friend of the people who alone is able to " smell out " the witch. He claims to be more radical than we are in not only dealing with the disease, but in getting rid of those who cause it. The witch is then looked upon as a kind of carrier! The poison ordeal frequently practised in some form or another is trial and punishment in one and the same act. Curiously enough the power of bewitching others is believed to have some connection with the possession of a "stomach." It may be that in the past, when inter-tribal warfare was common and cannibalism was the rule, man recognized that sometimes the stomach was distended and sometimes contracted. The former was deemed to be a mark of a witch, and it is a very much feared accusation to be told that one "has a stomach." It is often necessary to demonstrate this fact to those who come along not infrequently complaining that someone with a stomach has bewitched them. The "someone" is called and stands alongside his victim. It used to be my custom to produce four glasses, two pink and two blue. First of all the one accused would be instructed to drink a blue and a red draught and then to lie down on the ground to see if his belly swelled. Nothing would happen. The accuser would then drink his blue and red draughts, which contained in addition to coloured water a large dose of sodium bicarbonate in one and hydrochloric acid in the other. The interested onlookers would soon notice the outline of the incriminating organ and sometimes have audible proof of the existence of a "stomach"! The tables would then be turned and both accused and accuser would be sent away with the same stigma so that "the pot could not go on calling the kettle black."

It is, however, more than a joke. The terrible obsession of witchcraft is a dark shadow over Africa which accounts for more misery, social ostracism and ritual murder than anything else. It is only as the logical sequence of cause and effect can be demonstrated to the natives that they will cease to believe in it. The fear of witchcraft is a great force which can be taken hold of and sublimated into an effort at co-operation in hygiene and public health. Although so crude, it is much better than the fatalistic attitude, for it recognizes that somehow disease and premature death is athwart the plan of the universe and must be resisted and overcome.

But a handful of white doctors and nurses were quite inadequate for all these tasks. Our next object was the formation of a school for the training of native medical assistants. Boys with proficiency in French, the official language of the country, and mathematics were taken and indentured for a three years' course of practical work and theoretical study. Each does his appointed monthly task in the wards or clinic in the mornings and in the afternoon his time is devoted to lectures on anatomy and physiology, pharmacy, medical and surgical diseases. The native has not lost his powers of observation and an intensely practical course is the way to preserve this precious faculty in medicine. He is familiar with the appearances and accepted theories about current diseases, but very ready to exchange these for something more rational as revealed to him by the microscope. More especially is this the case if the disease is readily amenable to treatment as, are many tropical affections. He becomes an adept at the use of the intravenous injection, thousands of which are given every year. (Fig. 5.) A batch of young people from Bristol sent out an articulated skeleton for use in the classroom. This was carefully put together in the public gaze in order to prove that it came from Europe, and not from some victim. An old chief who eventually inspected it exclaimed with a chuckle: "Ha, a man with the meat off."

After three years' theoretical work the boys, if successful in their examination, are retained for another two years' practical work in the hospital or at a rural dispensary. They have by then learned to diagnose and treat common diseases, to recognize the ova of worms, blood parasites, etc., and are competent to give injections and to perform minor operations. A few who have shown themselves specially proficient have been encouraged to undertake a little major surgery, and one who eventually took up employment at a big oil plantation proudly showed me a ward full of cases of hernia, elephantiasis and hydrocele on which he himself had operated. He explained that the doctor who had come out from Europe was a young man who was afraid of the knife and had been only too willing to allow him to carry on for him. I believe he may have learned something from his assistant in so doing!

The government diploma is given after the fifth year and officially authorises the successful candidate to practise as an *infirmier diplômé*, but only under the supervision of a government, mission or commercial organization. This restriction of his practice is very necessary and wise, as those will testify who know conditions in India, where this class of assistant was formerly trained in large numbers but allowed private practice. This was frequently combined with quackery at its worst and failed entirely to meet the need of the rural population, for the assistant prefers to practise in the already overcrowded towns. In Africa a qualified native assistant has proved the most satisfactory solution to the rural problem where small clusters of people live in sparsely populated districts. Round the base hospital at Yakusu, in a district of 10,000 square miles, sixteen rural dispensaries now serve a population of 100,000. They are simple wattle and daub buildings, although permanent brick ones are now replacing some of the earlier ones. A waiting-room and dressing-room stand in front of a lock-up dispensary, which is stocked with standardized drugs and equipment, including a small zinc-top operating table. A stock of well-known, simple, yet effective drugs is always held for sale. This dispensary acts as a base for further rural extension work in the shape of native welfare clinics, which are often conducted by the *infirmiers'* wives, who have been trained at the base hospital.

One of the main reasons for the development of extension work as well as the intensive work of the hospital was the incidence in 1920 of a serious epidemic of sleeping sickness in the neighbourhood. Along the innumerable small waterways, along which canoes pass on fishing expeditions, and at shaded sites where women go to draw water, the tsetse fly (*Glossina palpalis*) is found in considerable numbers. This fly is so silent, its habit of approaching from behind so cunning, and the insertion of its proboscis so gentle, that it is frequently only the weight of its body distended with one's blood which first draws attention to its presence. An infected tsetse can fly from one to another and infect a number of people in a short space of time. The *Trypanosoma gambiense* develops in the foregut and within a fortnight has migrated from the body cavity to the salivary gland also situated in the abdomen. Both male and female tsetses are vectors.

The first symptom of infection, generally entirely ignored by natives, is a slight irritation round the bite with the production of a few vesicles. Trypanosomes may be found by a puncture of these. Later on a circinate rash on the chest is sometimes visible in white patients. Irregular fever is followed by a swelling of the lymphatic glands, most obvious in the neck, and the development of a persistent headache. The wearing of a fibre cord round the forehead is always a suspicious sign of sleeping sickness and the rather anxious look on the face is also typical.

The progress of the disease is relentless but slow. When the trypanosomes have found their way into the nervous system, as evidenced by their presence or an increased cell content of the cerebro-spinal fluid, temperamental changes occur. Frequently the patient becomes euphoric. Everything is a joke. You stick a lumbar puncture needle into his back and he roars with laughter. Others, however, have bouts of acute mania and have to be restrained. One such whom we had shut into a wood-store became violent as the full moon rose. One night he began throwing chunks of wood at the door, arousing the whole station. Using a small table as a shield, and with an assistant with a long forked stick behind me, I advanced slowly towards the victim, and when his store of ammunition had expended itself we got him pronged against the wall and eventually handcuffed to a heavy brick-machine.

Later on this mania gives place to complete apathy. Nothing arouses the interest, and finally twenty-two of the twenty-four hours are spent in sleep. The patient may actually doze off while in the act of raising food to the mouth. It is indeed pitiful to see a village containing a large number of patients of all ages and sexes in various stages of the disease. One of the first European patients whose case was studied was a lady who died in Bristol, and it was the examination of her brain which enabled Sir Frederick Mott to establish the characteristic pathology of the brain with the perivascular cuff of round cells which so resemble what one finds in G.P.I. It was fortunate that at the time this epidemic was detected we had received supplies of tryparsamide from the Rockefeller Institute for Medical Research in New York and a grant-in-aid for the investigation of its action. Its rapid curative effect in rabbits was hardly more spectacular than what occurred in human patients even in advanced stages of the disease. Constant visitation of the villages had to be undertaken for regular examination of every man, woman and child. (Fig. 6.) Some preferred to proceed to a treatment camp established near the hospital, but others received their weekly injections at treatment centres established in the endemic area. These latter were visited by boys who had all been trained as injectors, for the full *infirmier* course had not then been inaugurated. The treatment of sleeping sickness is not yet perfected: tryparsamide has the great drawback of causing blindness, and frequently one has to choose between that and death. In our own experience it has been only possible to cure about 50 per cent. of those well advanced in the third stage of the disease, and the reason why some respond and some do not is still an unsolved riddle, variously explained by resistant strain or by defective reactions. After eight years' work organized on these lines we were gratified to be able to state that not a single new case of sleeping sickness had occurred in the district, where formerly the incidence had been as high as 20 per cent.

There is very little time in the busy and many-sided life of the missionary for carrying out any systematic research work, especially as this is now the recognized task of a team of which a Mission Hospital can rarely

### PLATE VII

Fig. 5.
Medical assistants giving intravenous injection.

Fig. 6.
The doctor on tour in the district.

boast. We were fortunate, however, in receiving a grant from the Royal Society which enabled a Bristol graduate, Mr. A. C. Fisher, to proceed to Yakusu in 1933–34 and study our peculiar variety of intestinal schistosomiasis, the existence of which we had reported twelve years previously. By using white mice infected by a local species of snail he was able to study the morphology of what has now been admitted as a new species of Bilharzia worm named by him *S. intercalatum.* This trematode, as its name implies, lies half-way between *S. bovis* of cattle and the familiar *S. hæmatobium* of man. Its localization is, however, exclusively in the mesenteric veins, and the typical elongated terminal-spined ova are passed in the fæces. The symptoms are not so severe as in the case of the other human intestinal schistosomes *S. mansoni* and *S. japonicum,* but it causes a good deal of dysentery among children and non-immune adults. I have also clinical evidence that overflow of ova into the lungs produces broncho-pneumonic symptoms, and suspect occasional cerebral localization with serious results. The University of Bristol awarded Mr. Fisher an M.D. for this interesting piece of research.

Altogether the tremendous opportunities for medical and surgical work among primitive peoples and their responsiveness to one's efforts give one a profound sense of satisfaction in carrying on such work, and a feeling of utility as opposed to one of futility which must at times obsess us when dealing with more sophisticated folk. At a time when the whole medical profession of these islands is mobilized in an effort to succour those who may be the victims of injury deliberately inflicted it is a relief to turn to the proper task of the doctor, the relief of unmerited and s unnecessary suffering. It is even greater satisfaction to be able to demonstrate to people who firmly believe that all disease and death comes from spiritual or personal evil, and whose name for the Almighty was identical with that used for smallpox, that after all God is on the side of the angels.

NOTE.—All the illustrations are reproduced by permission of the Baptist Missionary Society.

## Figures and Tables

**Fig. 1. f1:**
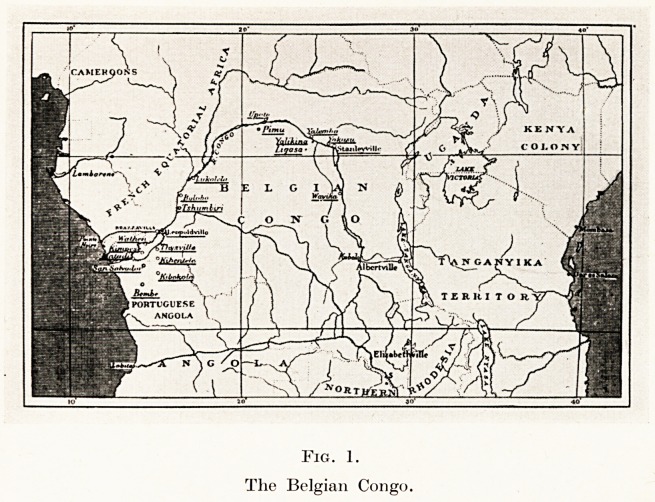


**Fig. 2. f2:**
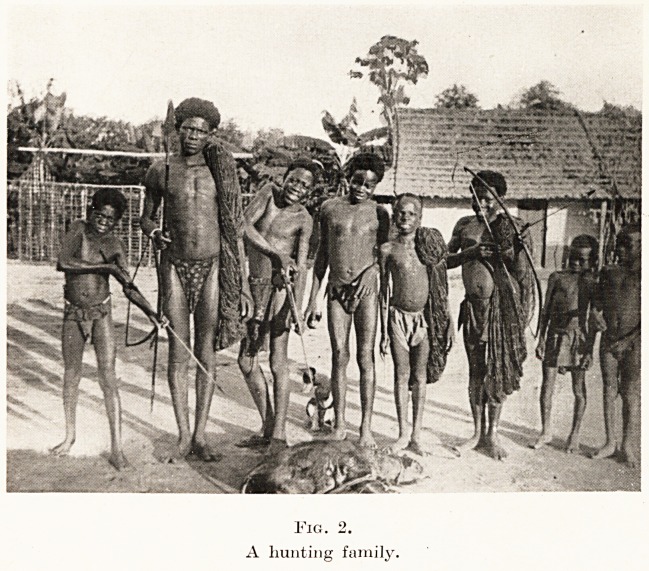


**Fig. 3. f3:**
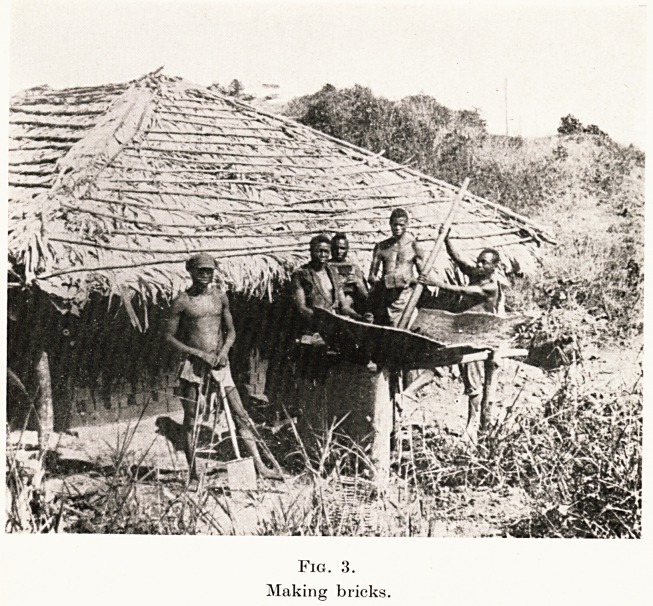


**Fig. 4. f4:**
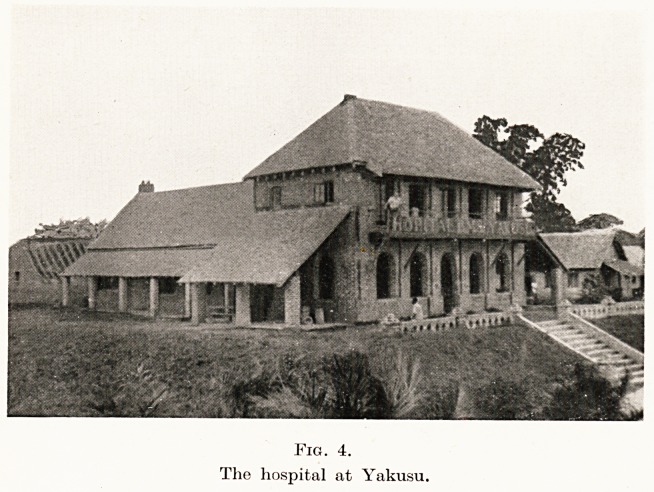


**Fig. 5. f5:**
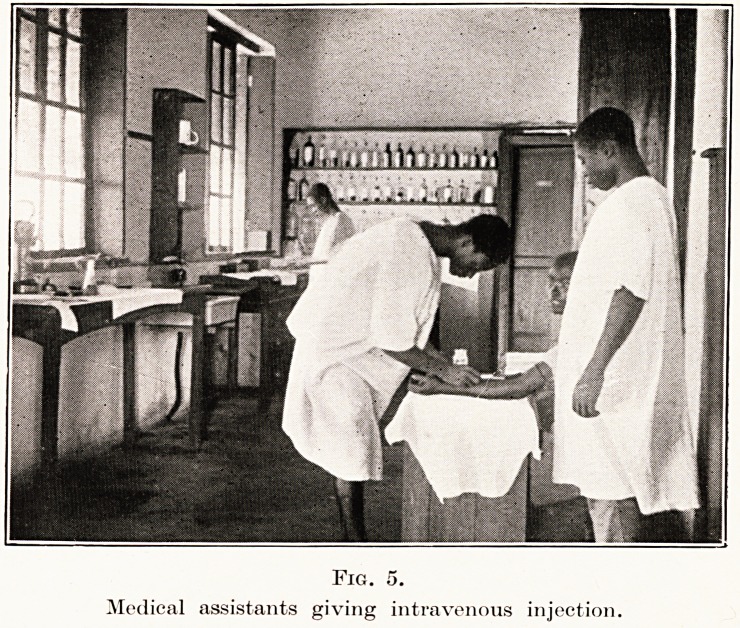


**Fig. 6. f6:**